# Sika Deer Antler Collagen Type I-Accelerated Osteogenesis in Bone Marrow Mesenchymal Stem Cells via the Smad Pathway

**DOI:** 10.1155/2016/2109204

**Published:** 2016-01-04

**Authors:** Na Li, Min Zhang, Gregor P. C. Drummen, Yu Zhao, Yin Fen Tan, Su Luo, Xiao Bo Qu

**Affiliations:** ^1^Center for New Medicine Research, Changchun University of Chinese Medicine, Changchun 130012, China; ^2^Medical Department, Beihua University, Jilin 132100, China; ^3^Cellular Stress and Ageing Program, Bionanoscience and Bio-Imaging Program, Bio & Nano-Solutions, 33647 Bielefeld, Germany

## Abstract

Deer antler preparations have been used to strengthen bones for centuries. It is particularly rich in collagen type I. This study aimed to unravel part of the purported bioremedial effect of Sika deer antler collagen type I (SDA-Col I) on bone marrow mesenchymal stem cells. The results suggest that SDA-Col I might be used to promote and regulate osteoblast proliferation and differentiation. SDA-Col I might potentially provide the basis for novel therapeutic strategies in the treatment of bone injury and/or in scaffolds for bone replacement strategies. Finally, isolation of SDA-Col I from deer antler represents a renewable, green, and uncomplicated way to obtain a biomedically valuable therapeutic.

## 1. Introduction

Sika deer velvet, especially the dried immature velvet antler (VA) of* Cervus nippon Temminck*, produced in Jilin Province of China, is a precious ingredient used in traditional Chinese medicine (TCM). A major biological characteristic of VA is its rapid growth, and consequently various growth factors related to growth of cartilage, bone, epidermis, and other tissues are abundantly expressed or show increased biological activity [1–3]. In various parts of Asia, including China, deer antler has been used for centuries in TCM and clinical observations from Asia convincingly showed that VA preparations strengthen bones and muscles, promote blood flow, reduce chronic joint pain, reduce gynecological derangements, positively affect antioxidative capacity, reduce cholesterol levels, and increase immune function, to name but a few [2–4]. Furthermore, recent scientific investigations have shown that deer antler preparations were able to reduce the side-effects of cancer treatment, promote chondrocyte and osteoblast precursors proliferation, and reduce oxidative DNA damage in lymphocytes and that deer antler preparation aqua-acupuncture showed antibone resorption in adjuvant-induced arthritic rats [5–8]. Because harvesting VA from deer is uncomplicated and painless and carried out in compliance with a strict Velveting Code of Practice under veterinary supervision, VA production through deer farming is an attractive and sustainable green source for the isolation of biologically active components for medicinal purposes, provided that these and their biological and bioremedial properties are known and fully understood.

Deer antler is similar in composition to human bone and consists of a calcium phosphate matrix of hydroxyapatite (73%), with smaller amounts of calcium carbonate, and 27% organic material [9]. Therefore, the bone strengthening effect of deer antler purported in TCM might be by acting as a calcium source in the treatment of bone degeneration; hydroxyapatite is considered to be the form of calcium that is most efficiently absorbed. The organic matrix on the other hand is particularly rich in collagen type I (SDA-Col I) content (80–90%). In humans, Col I is the main constituent of fibrous tissues and can be found in the cornea, skin, arterial walls, tendons, myofibrils, fibrocartilage, and bones and teeth and is the most abundant collagen type and extracellular matrix (ECM) protein [10]. Structurally, Col I forms a heterotrimer consisting of two *α*_1_-chains and one *α*_2_-chain (*γ* triple helix), encoded by* COL1A1* (MIM: 120150) and* COL1A2* (MIM: 120160) [10, 11]. In its precursor form, procollagen type I, the structure consists of a central helical domain (repeating [Gly-X-Y] triplet), flanked by an amino- (N-) and carboxy- (C-) terminal propeptide, is synthesized and folded in the rough endoplasmic reticulum, and subsequently transported and secreted into the ECM [10, 12, 13]. After cleavage of the N- and C-terminal propeptide by specific proteinases, mature collagen is formed and undergoes self-assembly into fibrils. In bone, the collagen triple helices are oriented in parallel, staggered arrays with 40 nm gaps between the ends of the subunits that most likely serve as nucleation sites for crystals of the mineral hydroxyapatite component of bone. Consequently, Col I plays a key role in maintaining the structural integrity and biomechanical properties (tensility and ductility) of bone [14]. However, it is increasingly becoming clear that beyond these structural functions, Col I and/or its propeptides are involved in signaling events, cell recruitment, and cell differentiation, amongst others. For instance, Col I is able to stimulate angiogenesis* in vitro* and* in vivo* through ligation and most likely clustering of endothelial cell surface *α*_1_ *β*_1_/*α*_2_ *β*_1_ integrin receptors by specific sequences in the collagen fibrils [15]. It has been well documented that preosteoblast grown on Col I films shows a temporally early and enhanced expression of the osteoblast phenotype compared with cells grown on other surfaces [16, 17]. In particular, work by Suzawa and coworkers has shown that collagen-elicited focal adhesion kinase (FAK) and extracellular signal-regulated kinase (ERK) signaling congregate with bone morphogenetic protein (BMP) signaling at the level of SMAD activation in the nucleus [18]. Furthermore, positive effects of oral intake of collagen or collagen hydrolysate on osteoarthritis have been reported [19, 20]. In addition to these fundamental studies on Col I, circulating levels of the carboxy-terminal propeptide of Col I in deer have been shown to increase during antler regeneration [21].

Because of the aforementioned factors, we aimed to investigate if the purported bioremedial and bone strengthening effects of SDA-Col I might be a result of osteogenesis induction and if so, by what mechanism. To this end we used bone marrow mesenchymal stem cells (BMSCs) as a model system, since these have been shown to be able to differentiate into a variety of cell types, including osteoblasts, chondrocytes, adipocytes, and neural cells [22], and can be induced to enhance bone formation. We provide evidence that SDA-Col I not only is extending the lifespan of osteogenetic cells, but also accelerates osteogenetic differentiation of BMSCs through enhanced cell cycle progression, as well as activation of osterix and the Smad pathway.

## 2. Materials and Methods

### 2.1. Sika Deer Antler Collagen Type I Isolation

Collagen type I was extracted from Sika deer antler (kind gift from Professor Zhao Yu (Center for New Medicine Research, Changchun University of Chinese Medicine)) via a trypsin hydrolysis method. Briefly, whole fresh deer antler was obtained from sika deer (Cervus Nippon Temminck) produced in Jilin Province of China. The raw materials (1 kg) were cut into small pieces (approximately 1 cm^3^) and washed with cold distilled water (approximately 4°C) to remove blood. The collagens of SDA were extracted by the traditional collagen abstract method [1]. The pretreated sample of SDA was mixed with 0.1 M NaOH at a solid to alkali solution ratio of 1 : 10 (w/v) for 24 h. Then, the sample was washed with cold water until the neutral or faintly basic pH of wash water was obtained. The SDA hydrolysate was prepared using enzymes hydrolysis condition for trypsin enzymolysis for 2 h at 37°C. To stop the reaction, the mixture was heated immediately at 100°C for 20 min and then centrifuged at 4000 ×g for 30 min, and the supernatant was freeze-dried. The powder of the water-solubilized extract was collagen peptide. Protein concentration was measured by Lowry method using a protein assay kit (Beijing Dingguo Biotech. Co., Ltd.) that was around 56.89% [2].

### 2.2. Amino Acid Analysis

Collagen Z was hydrolyzed under vacuum in 6 mol/L HCl for 24 h at 110 ± 1°C for general amino acid analysis. Amino acids converted to phenyl isothiocyanate (PITC) derivatives were analyzed by high performance liquid chromatography (HPLC) (Agilent 1100 series; Agilent Technologies, USA) with Wondasil-C18 (4.6 × 150 mm, 5 *μ*m). Eluents were 97% aqueous acetonitrile containing 0.1 mol/L sodium acetate (pH6.5, eluent A) and 80% aqueous acetonitrile (eluent B); flow rate was 1.0 mL/min; the detection wavelengths were at 254 nm and the column temperature was 37°C. The gradient elution procedures are ignored.

### 2.3. Cell Culture

12-week-old male Sprague-Dawley rats with an average weight of 250 ± 15 g were obtained from the Jilin University Animal Center (Changchun, China). All animal experimental procedures were approved by the Animal Care and Experiment Committee of Changchun University of Chinese Medicine, and BMSCs were isolated and cultured according to the protocol reported previously [22]. Briefly, both ends of the femora were cut off at the epiphysis and bone marrow was flushed out with HyClone Minimum Essential Medium (MEM; Thermo Fisher Scientific, Waltham, MA, USA) supplemented with 10% fetal bovine serum (FBS; Gibco/Life Technologies, Grand Island, NY, USA), 100 IU/mL penicillin, 100 IU/mL streptomycin, and 2 mmol/L L-glutamine in a humidified atmosphere (5% CO_2_, 37°C) [23]. Cells were counted in suspensions using a Cedex analyzer (Innovatis AG, Bielefeld, Germany). Cell surface molecules CD29, CD34, and CD45 (1 × 10^4^ cells) were analyzed and stained with CD29-FITC, CD34-FITC, and CD45-FITC staining kit (Kaiji Bio Co., Nanjing, China) according to the manufacturer's instructions. Cells were immediately analyzed by flow cytometry on a FACSAria flow cytometer (Becton Dickinson Biosciences, San Jose, CA, USA).

### 2.4. Osteogenic Induction

For osteogenic differentiation, BMSCs were seeded in six-well plates at an initial density of 5 × 10^4^ cells/well and maintained in MEM, as described above. After reaching subconfluency, cells were treated with osteogenic induction medium for 14 days, containing SDA-Col I in the range of 0.5–10 *μ*g/*μ*L (SDA-Col I group), or 10 nM dexamethasone (Dex group; Sigma-Aldrich, St, Louis, MO, USA), or medium only (control group) [24]. The osteogenic induction medium consisted of MEM supplemented with 10% FBS, 50 *μ*M L-ascorbic acid 2-phosphate (Sigma-Aldrich), and 10 mM *β*-glycerophosphate (Sigma-Aldrich). Cells were kept in culture and the medium was replaced every 3 days.

### 2.5. Cell Viability Assay

Cell proliferation and concomitantly SDA-Col I cytotoxicity were assessed via the MTT (3-(4,5-dimethylthiazol-2-yl)-2,5-diphenyltetrazolium bromide) assay, which is based on the reduction of a yellow soluble tetrazole to an insoluble purple formazan in respiring cells. Cells were seeded at an initial density of 5 × 10^4^ cells/well in a 96-well plate and treated with 0.5, 1.0, 2.5, 5.0, and 10.0 *μ*g/*μ*L SDA-Col I for 4, 7, and 14 days. At the end of the treatment, media containing SDA-Col I were carefully aspirated and 200 *μ*L medium with 20 *μ*L of a 5 mg/mL MTT (Sigma-Aldrich, St. Louis, MO, USA) solution in PBS was added to each well. After 4 h of incubation at 37°C, the medium was removed and 100 *μ*L DMSO was added to each well. The optical absorbance (A) was measured at 490 nm using a BioTek ELX800 multiwell reader (BioTek, Winooski, VT, USA) [25]. The percentage of viable cells (VCs) was calculated according to

(1) $$\begin{array}{c} CV\left(\;\%\;\right)=\left(\;\frac{\text{A of experimental group}}{\text{A of control group}}\;\right)\times 100\%. \end{array}$$

### 2.6. Flow Cytometric Analysis of Cell Cycle in BMSCs

BMSCs were seeded in 6-well plates (4 × 10^5^ cells/well) and treated for 14 days with the various compounds at the indicated concentrations. Cell cycle analysis was performed via flow cytometry on a FACSAria flow cytometer (Becton Dickinson Biosciences) after propidium iodide (PI) staining. Briefly, treated and untreated cells were detached with 0.05% trypsin for 3 min at room temperature, fixed with ethanol at 4°C for 4 h, and then stained with 50 *μ*g/mL of PI (100 *μ*g/mL RNase A) in PBS. The resulting cell suspensions were immediately analyzed by flow cytometry and the cell cycle phase was determined in each sample with ModFit LT software (Verity Software House, Topsham, ME, USA).

### 2.7. Assessment of Alkaline Phosphatase

After SDA-Col I treatment of 14 days, cells were stained using the alkaline phosphatase (ALP) Color Development Kit (Beyotime Institute of Biotechnology, Haimen, Jiangsu, China) according to the manufacturer's protocol. Images were recorded on an Olympus BX51TRF transmitted and reflected light research microscope with a photomicrograph system (Olympus Inc., Tokyo, Japan).

### 2.8. Oil-Red-O Staining of Differentiated MSCs

In this study, the BMSCs were cultured in osteogenic differentiation medium for 14 days. Oil-red-O staining was used to assess the accumulation of lipid droplets in differentiated adipocytes from BMSCs. BMSCs were washed with PBS and fixed by 2% neutral buffered formalin for 20 minutes, and cells were washed with distilled water and again washed with 60% isopropanol. Cells were stained with a fresh 60% Oil-Red-O staining (0.5 g Oil-Red-O in 100 mL isopropanol) for 15 min and washed with 60% isopropanol and again with distilled water. Imaging was performed via optical microscopy at room temperature.

### 2.9. Real-Time PCR

ALP, OC, Smad2, Smad3, Smad4, and *β*-actin (housekeeping control) PCR primers were designed using Primer Express software (Perkin-Elmer Biosystems, USA) based on their published sequence (Table 1). Total RNA from BMSCs was isolated with TRIzol reagent (Invitrogen/Life Technologies, Grand Island, NY, USA) according to the manufacturer's instructions. To avoid DNA contamination, total RNA was treated with RNase-free DNase I (Takara, Kyoto, Japan) for 60 min at 37°C and extracted once again with the TRIzol reagent. The RNA purity was determined spectrophotometrically from the 260/280 nm absorbance ratio and the RNA integrity was assessed by determining the intensity of the 28S and 18S rRNA bands after formaldehyde agarose gel electrophoresis. Total RNA (2 *μ*g) was subjected to reverse transcription using a RevertAid First-Strand cDNA Synthesis Kit (Fermentas Inc., Glen Burnie, MD, USA) with a random hexamer primer, and 2 *μ*L cDNA solution was subsequently used for real-time PCR. Genes were amplified in a 25 *μ*L reaction volume using SYBR Green (Applied Biosystems, Forster City, CA, USA) on a MiniOpticon Real-Time PCR System (Bio-Rad, Hercules, CA, USA).

The temperature profile consisted of an initial denaturation step at 95°C for 5 min, followed by 40 cycles at 95°C for 10 s, 58°C for 15 s, and 72°C for 10 s before melting curve analysis. The specificity of the amplified products was evaluated via agarose gel electrophoresis and was further verified with automated cycle sequencing. To ensure consistency in threshold cycle (Ct) values, duplicate reactions were performed and mean Ct values were used for calculating the relative expression levels. The Ct values were analyzed as described previously by Zhou et al. [26] and the normalized Ct values for each gene were subjected to Student's *t*-test with two-tailed distribution to determine statistical significance (95% confidence interval). Reactions were carried out in triplicate and the mean value was used. For standardization, *β*-actin was used as an internal control of each sample.

### 2.10. Western Blotting

After treatment with SDA-Col I, BMSCs were washed twice with precooled PBS, 10^6^ BMSCs were washed twice with precold PBS and treated with RIPA buffer (50 mmol/L Tris (pH 8.0), 150 mmol/L NaCl, 0.1% SDS, 1% NP40, and 0.5% sodium deoxycholate) containing protease inhibitors (1% Cocktail and 1 mmol/L PMSF). Total proteins were separated by 15% SDS-PAGE and transferred to PVDF membranes. The membrane was blocked for with Tris-buffered saline with 0.1% Tween 20 (pH 7.6, TBST) for 1 h at room temperature and the PVDF membrane was immunoblotted with first antibody solution (1 : 1000) at 4°C overnight. After washing twice with TBST, the membrane was incubated with HRP-labeled secondary antibody (Santa SC-2073) for 1 h at room temperature and washed three times with TBST. Final detection was performed with enhanced chemiluminescence (ECL) Western blotting reagents (Amersham Biosciences, Piscataway, NJ) and membranes were exposed to Lumi-Film Chemiluminescent Detection Film (Roche) [27]. Loading differences were normalized using a monoclonal *β*-actin antibody. The primary antibodies used in this study included anti-ALP (SC-98652), anti-OC (SC-365797), anti-BMP-2 (SC-137087), anti-Smad2 (SC-101153), anti-Smad3 (SC-7960), anti-Smad4 (SC-7966), and anti-*β*-actin (SC-130301) and were all acquired from Santa Cruz Biotechnology, Inc. (Santa Cruz, CA, USA).

### 2.11. Statistics and Data Analysis

Measurements were performed in triplicate and results are expressed as a means ± SD. SDs in fold change were calculated using the propagation of error formula. Data were obtained from at least three independent experiments. Analysis of variance (ANOVA) for multiple comparisons was carried out using statistical analysis software (SPSS, Chicago, IL, USA). In all cases, values of *P* below 0.05 were considered to indicate significant differences [28].

## 3. Results 

### 3.1. Molecular Weight and Amino Acid Contents

**Figure 1:**
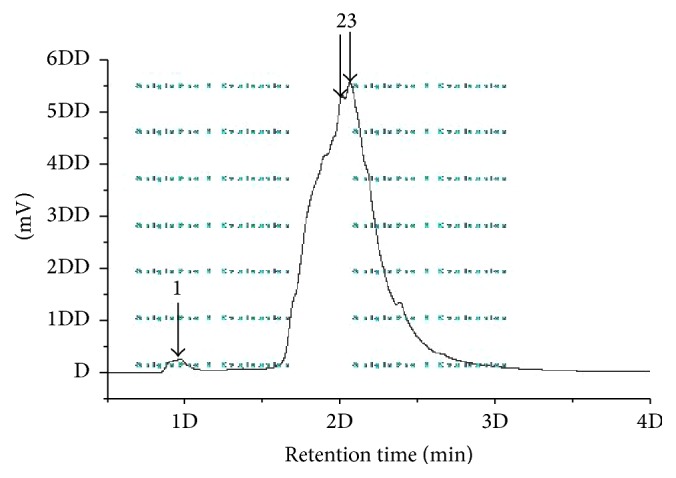
HPLC profiles of collagen I of SDA. Collagen I was treated with trypsin (1 : 1000, w/w) at 37°C for 3 h after boiling water.

HPLC gel filtration chromatography was used to evaluate the molecular weight. HPLC profile of collagen I of SDA was shown in Figure 1. There was a linear relation between the retention time and the molecular mass of the reference proteins in the range of 9.137–15.736 kDa (the retention time of a peak in the HPLC of the sample is about 20–30 min, regression equation: *y* = −0.0788*X* + 21.36, *r* ^2^ = 0.9951). Molecular size distributions with HPLC of collagen I were showed in Table 2, and the total collagen peptide was about the 9–15 kDa.

As shown in Table 3, amino acid compositions of SDA had Gly as the major amino acid which is the most dominant amino acid in collagen [2, 3]. The collagen peptide contained seven essential amino acids: lysine, leucine, phenylalanine, threonine, valine, isoleucine, and methionine. Glycine (32.102%) was the major component in the amino acids of collagen peptide. Proline and hydroxyproline accounted for 15.500% and 6.709% of the total amino acid residues, respectively. Tyrosine, methionine, and histidine were the lowest in the amino acids of collagen peptide.

### 3.2. Identification of BMSCs

**Figure 2:**
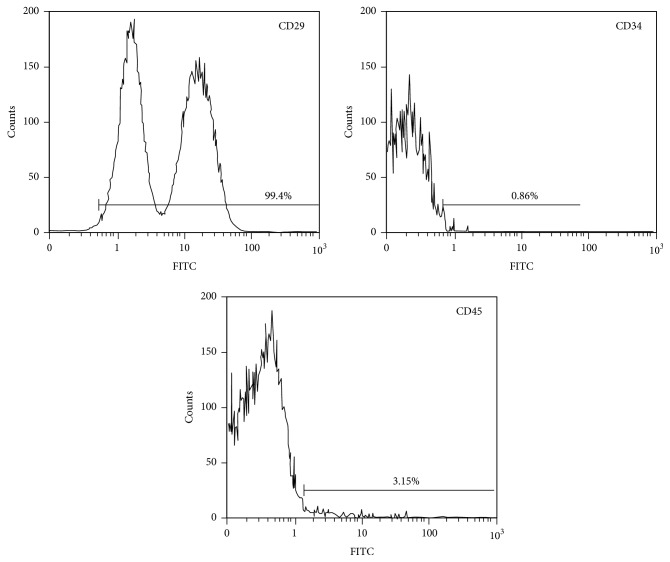
Flow cytometric identification and evaluation of lineage differentiation potential of bone marrow mesenchymal stem cells (BMSCs). BMSCs identification with surface markers: CD29(+), CD34(−), and CD45(−).

To ensure an accurate evaluation of cellular effects, and the biocompatibility and possible osteoinductivity of SDA-Col I, BMSCs were isolated as previously described, and lineage specific surface markers were evaluated to determine lineage and differentiation potential. The isolated cells expressed the MSC marker CD29 but were negative for the hematopoietic marker CD34 and the leukocyte marker CD45 (Figure 2). These results were in concordance with the BMSCs population phenotypic characteristics described in previous research [29, 30].

Dexamethasone (Dex), a potent synthetic member of the glucocorticoid class of steroid drugs, was used as a positive control, since Dex has been shown to enhance the osteogenic differentiation potential of mesenchymal stem cells [31, 32]. We used 10 nM Dex, because higher concentrations suppress osteogenic differentiation and induce a shift to adipogenic differentiation [33, 34], and low-dose Dex maintains the cell-surface phenotype [35]. Furthermore, high doses of Dex reduced the proliferative capacity of BMSCs, as shown in Figure 2 (first column; ~75%).

### 3.3. Effects of SDA-Collagen Type I and Dexamethasone on Cell Proliferation

**Figure 3:**
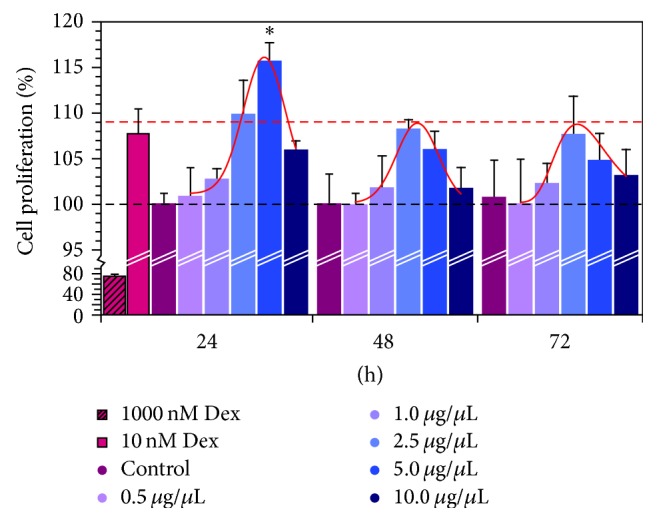
Assessment of cell proliferation of bone marrow mesenchymal stem cells (BMSC) via the MTT assay. BMSCs were seeded at an initial density of 5 × 10^4^ cells/well and grown in the presence of various concentrations SDA-Col I. All data are means ± SD from triplicates (*n*⩾3); ^*∗*^ *P* < 0.05.

The effects of SDA-Col I and dexamethasone treatment on the proliferative capacity of the BMSCs and concomitantly cytotoxicity of SDA-Col I were determined based on the capacity of the BMSCs to proliferate (MTT assay). Following 4, 7, and 14 days of treatment with different concentrations of SDA-Col I, no significant cytotoxic effects were observed for the concentrations used. On the contrary, SDA-Col I was able to induce BMSC proliferation in a dose-dependent manner; the maximum effect occurred around 2.5–5.0 *μ*g/*μ*L (Figure 3). Furthermore, 14 days of treatment produced the largest proliferative effect and compared with control cells, 5.0 *μ*g/*μ*L SDA-Col I increased BMSC proliferation 1.16 ± 0.02-fold, still 1.07 ± 0.03-fold larger than 10 nM Dex. The fit profiles in Figure 3 also indicate that this dose-dependence remained. Overall, SDA-Col I treatment produced time and dose-dependent BMSC proliferation with the maximum proliferative effect at 14-day treatment and 5.0 *μ*g/*μ*L SDA-Col I.

### 3.4. Flow Cytometric Analysis of Cell Cycle

**Figure 4:**
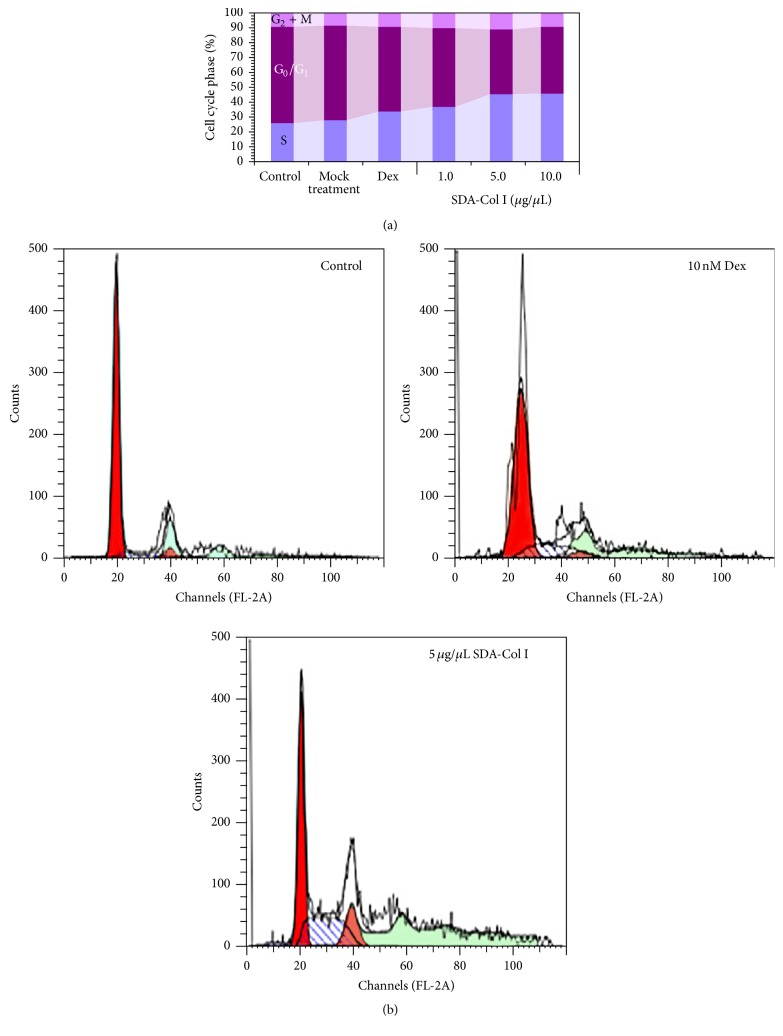
Cell cycle analysis (representative results) of SDA-collagen type I-treated bone marrow mesenchymal stem cells (BMSC). (a) BMSCs were seeded at an initial density of 4 × 10^5^ cells and grown for 14 days in the presence of various concentrations SDA-Col I or 10 nM dexamethasone (Dex), after which the cell cycle phase distributions were determined via flow cytometry. (b) Representative cell distribution profiles from flow cytometry.

As shown above, SDA-Col I treatment was able to significantly enhance cell proliferation. To further assess this positive effect, the effect on cell-cycle phase distribution after 14 days of treatment with SDA-Col I was determined (Figure 4) by analyzing DNA content. In control BMSCs, cell populations in the G_0_/G_1_, S, and G_2_ + M phases were 25.9 ± 1.02%, 65.2 ± 0.98%, and 8.9 ± 1.01%, respectively. Mock treatment did not significantly affect this distribution, whereas incubation with 10 nM Dex increased the S phase 1.3-fold (25.9→33.7%) compared with control. Conversely, 5 *μ*g/*μ*L SDA-Col I increased the S phase population to 45.6% (~1.8-fold) and reduced the G_0_/G_1_ phase population to 44.7% (~1.5-fold) compared with control cells. No significant change in cell cycle phase distribution was observed at higher concentrations SDA-Col I, which was in agreement with the cell proliferation measurements (Figure 4). Collectively, these observations suggest that treatment with SDA-Col I affected the cell cycle and significantly upregulated the S phase.

### 3.5. Alkaline Phosphatase and Osteocalcin Expression in BMSCs Treated with SDA-Collagen Type I

**Figure 5:**
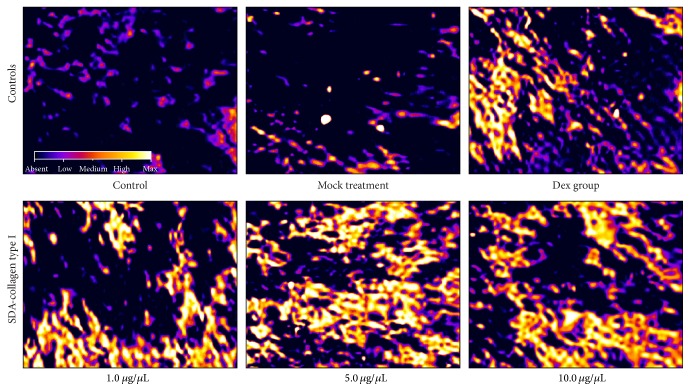
Microscopic evaluation of the osteoplastic differentiation-related marker alkaline phosphatase (ALP) in bone marrow mesenchymal stem cells (BMSCs) in response to 14-day SDA-Col I treatment or 10 nM dexamethasone (Dex). Light microscopic assessment of ALP expression in a BMSC cell population (ALP Color Development Kit). Lineage differentiation: osteoblasts (ALP staining), cytoplasm (Blue), nucleus (Pink), and ALP expression in a BMSC cell (Yellow). Bar: 20 *μ*m.

Since alkaline phosphatase (ALP) and osteocalcin (OC) are early osteogenic differentiation markers, we determined the effect of SDA-Col I on ALP and OC expression by culturing BMSCs on 5.0 *μ*g/*μ*L SDA-Col I in osteoplastic medium; OC is a small vitamin K-dependent protein that is exclusively synthesized in osteoblasts and megakaryocytes [36]. An initial microscopic assessment of ALP expression in a BMSC cell culture, as shown in Figure 5, revealed that the ALP staining increased with increasing concentration of SDA-Col I and that this effect was generally larger at SDA-Col I concentrations above 5.0 *μ*g/*μ*L compared with the Dex group; ALP expression at 1.0 *μ*g/*μ*L SDA-Col I was nearly equal to ALP expression at 10 nM Dex (Figure 5). Fluorescence microscopic observation of individual cells (Figure 5) stained with anti-ALP antibody showed characteristic granular or punctuated structures on the cell surface and, with increasing concentration SDA-Col I, increased staining in distinct regions of the plasma membrane. At SDA-Col I concentrations above 5.0 *μ*g/*μ*L, increasingly staining of both the granular structures and the plasma membrane was observed. No significant differences were observed at higher concentrations SDA-Col I, which was in good agreement with the results in Figure 5 and the results from the cell proliferation and cell cycle analyses. Alkaline phosphatase is a surface-bound enzyme anchored to the plasma membrane through a phosphatidyl inositol-glycophospholipid (GPI) anchor, covalently attached to the C-terminus of the enzyme [37]. The observed punctuated staining pattern might be due to local differences in enzyme activity, and as differentiation progresses, ALP pools become increasingly activated, which results in more uniform plasma membrane staining. Finally, notice the overall absence of formation of long cellular projections, but normal cell spreading.

**Figure 6:**
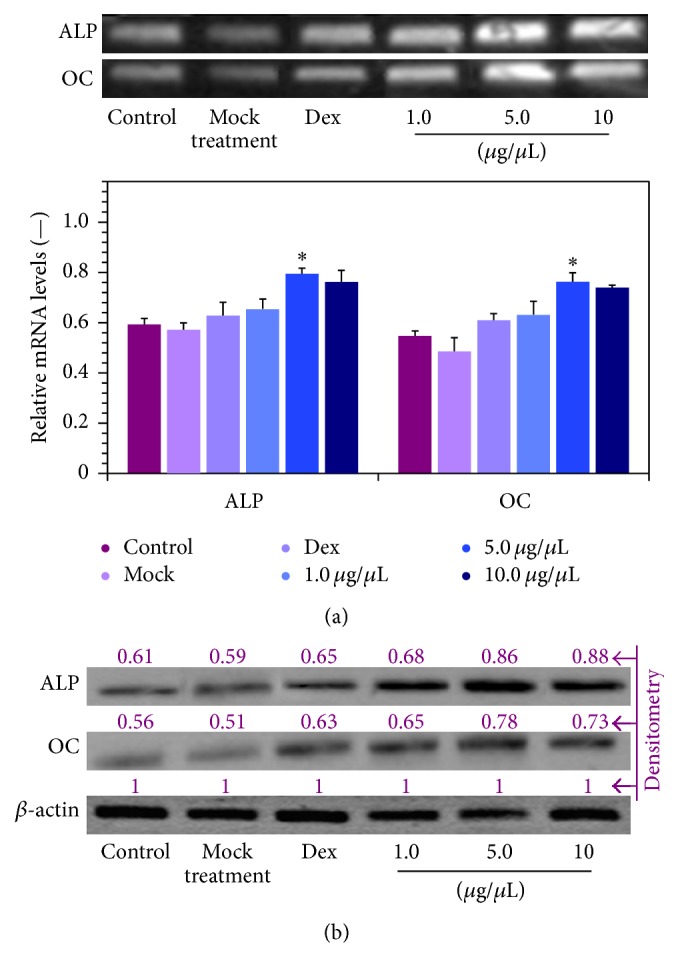
Effect of SDA-collagen type I on the expression of alkaline phosphatase (ALP) and osteocalcin (OC) in bone marrow mesenchymal stem cells (BMSCs) after 14-day treatment (in osteogenic induction medium). (a) mRNA expression as assayed via real-time PCR. Data shown are normalized means + SD relative to *β*-actin mRNA levels (*n*⩾3). *∗* denotes a significant difference (*P* < 0.05). (b) Representative Western blots of ALP and OC protein expression (*n*⩾3). Results were quantified via densitometry with the maximum grey value set to 1. All blots were run under the same experimental conditions.

Since the aforementioned microscopy results showed that SDA-Col I could induce ALP expression, we measured changes in ALP and OC mRNA and protein expression to obtain a more quantitative evaluation of a possible SDA-Col I-induced osteogenesis. To this end, real-time PCR and Western blotting analyses were used to detect the expression of target genes in response to various concentrations SDA-Col I. The results in Figures 6(a) and 6(b) show that SDA-Col I was able to induce ALP and OC mRNA and protein expression. These effects were concentration dependent and showed a similar pattern to the proliferation measurements in Figure 3; albeit that at the highest concentration SDA-Col I, osteogenesis is favored over proliferation. The maximum effect was achieved at 5.0 *μ*g/*μ*L SDA-Col I for both ALP and OC, which resulted in an increase in mRNA levels by a factor of 1.34 ± 0.10 and 1.38 ± 0.09 compared with control, respectively. At higher concentrations SDA-Col I (>10 *μ*g/*μ*L), ALP mRNA expression was suppressed and reduced to approximately control levels, whereas OC mRNA levels were only marginally increased compared with control cells. Similar effects were observed on Western blot, with ALP and OC protein levels being increased by a factor of 1.44 ± 0.22 and 1.39 ± 0.12 in response to 5.0 *μ*g/*μ*L SDA-Col I.

**Figure 7:**
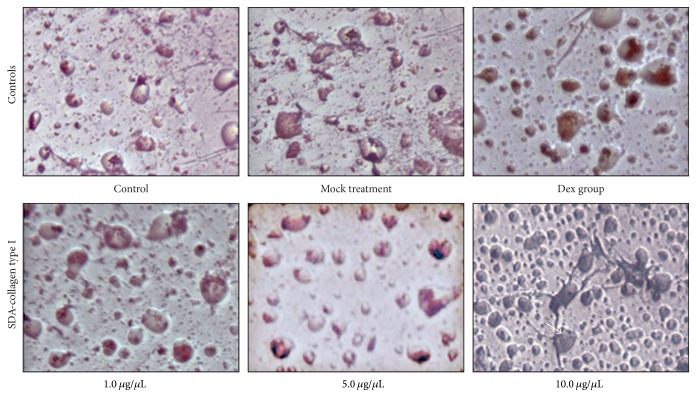
Microscopic evaluation of adipogenic differentiation in bone marrow mesenchymal stem cells (BMSCs) in response to SDA-Col I treatment or 10 nM dexamethasone (Dex). Cells were stained with Oil Red O to determine lipid droplet formation as a measure for adipogenesis.

To determine if treatment with SDA-Col I induced adipogenic differentiation, BMCs were stained with Oil Red O to evaluate lipid droplet formation. The results in Figure 7 show that some level of lipid droplet formation is observable in the control, mock, and Dex treated groups, and no significant deviation occurred up to 5 *μ*g/*μ*L SDA-Col I. Only at 10 *μ*g/*μ*L SDA-Col I was an increase in lipid droplet formation noticeable, which indicates that at higher concentrations, SDA-Col I induces adipogenic differentiation to some extent.

### 3.6. SDA-Collagen Type I-Accelerated Osteogenesis through Smad Pathway Activation in BMSCs

The bone morphogenic protein (BMP)/Smad signaling pathway plays an important role in the proliferation and differentiation of osteoblasts [38, 39]. We therefore determined the effect of SDA-Col I treatment on the activation of BMP2 and Smad proteins. Since Smad4 is the only Smad involved in both transforming growth factor beta (TGF-*β*) and BMP signaling, we included Smad4 in our evaluation. As shown above, SDA-collagen type I treatment induced ALP and OC expression (Figure 6) and accelerated osteogenesis.

**Figure 8:**
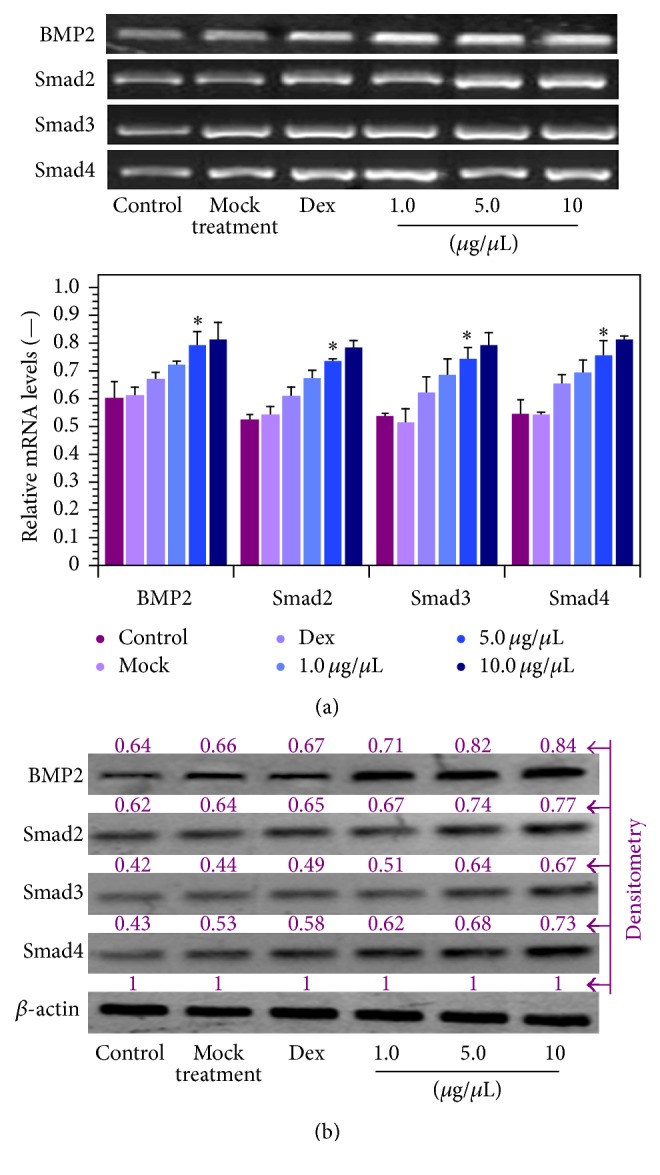
Effect of SDA-collagen type I on the expression of BMP2, Smad2, Smad3, and Smad4 in bone marrow mesenchymal stem cells (BMSCs) after 14-day treatment (in osteogenic induction medium). (a) mRNA expression as assayed via real-time PCR. Data shown are normalized means + SD relative to *β*-actin mRNA levels (*n*⩾3). *∗* denotes a significant difference (*P* < 0.05). (b) Representative Western blots of BMP and Smad protein expression (*n*⩾3). Results were quantified via densitometry with the maximum grey value set to 1. All blots were run under the same experimental conditions.

The results in Figure 8 show that SDA-Col I treatment (14 days) resulted in the induction of BMP2 and Smad2, Smad3, and Smad4 proteins, as deduced from the increase in mRNA and protein expression. These effects were concentration dependent and the maximum effect was obtained with 10 *μ*g/*μ*L SDA-Col I. Compared with the control group, 5 *μ*g/*μ*L SDA-Col I was able to increase the mRNA expression of BMP2, Smad2, Smad3, and Smad4 (Figure 8(a)) by a factor of 1.32 ± 0.17, 1.40 ± 0.04, 1.38 ± 0.08, and 1.38 ± 0.16, respectively. Conversely, 10 nM Dex increased BMP2 and Smad levels only by a factor of 1.11 ± 0.12, 1.15 ± 0.07, 1.15 ± 0.11, and 1.17 ± 0.12, respectively, whereas mock treatment had no significant effect. Protein levels of BMP2, Smad2, Smad3, and Smad4 (Figure 8(b)) increased on average 1.29 ± 0.12-, 1.26 ± 0.08-, 1.42 ± 0.11-, and 1.51 ± 0.12-fold, respectively. Equally, mock treatment showed virtually no effect and 10 nM Dex increased BMP2 and Smad levels on average ~1.15-fold. These results suggest that SDA-Col I-accelerated osteogenesis involved the activation of the BMP2/Smad pathway.

## 4. Discussion

Bone cells originate from mesenchymal stem cells, which, through correct signaling events, develop into osteoblasts and eventually osteocytes. The initial process involves recruitment of osteoblast progenitor cells to the bone surface and succeeding proliferation and differentiation into mature osteoblasts. These in turn mineralize the extracellular matrix. Nonetheless, the exact mechanisms, especially microenvironmental cues, involved in bone formation and replacement as a consequence of injury are multifactorial and complex. Novel therapeutics and materials and substrate materials that allow stimulation of bone formation and may serve as scaffolds for bone tissue engineering in surgical replacement strategies are desperately sought after. Collagens have previously been shown not only to play an important role in the structural integrity of bone, but also to positively affect bone formation and replacement mechanisms.

Traditionally, Sika antler, especially the dried immature velvet antler, has been used extensively in Asian medicine for a myriad of ailments. In particular, positive effects from deer anther preparations have been observed on derangements involving bone or cartilage during centuries of clinical observations. A major advantage of using VA preparations for biomedical applications is the fact that production through deer farming is uncomplicated and represents a renewable, green, and economically attractive source of potentially bioactive compounds. However, if such preparations are to be used for medical purposes, their bioremedial efficacy should be supported by solid scientific evidence.

Here we show that the presence of SDA-Col I positively affects BMSC proliferation and differentiation in a concentration dependent manner. Although SDA-Col I increased the proliferative capacity of BMSCs by nearly 20%, with the maximum effect at 5.0 *μ*g/*μ*L SDA-Col I, this positive proliferative effect was attenuated both at higher concentrations SDA-Col I and as time increased; albeit that proliferation always remained higher than in untreated control cells. Similar effects have been reported for Dex treatment, although partially different mechanisms of action are likely to be involved (integrin receptor/BMP/Smad signaling versus downstream nuclear steroid receptors). Both high concentrations of Dex and prolonged treatment were shown to reduce the proliferative capacity and induce osteoblast commitment and maturation [31]. In fact we confirmed this concentration dependent attenuation of BMSC proliferation, since in a 14-day concentration series, proliferation dropped to ~91% at 100 nM Dex (not shown) and nearly 75% at 1000 nM (Figure 2). This increased proliferation under SDA-Col I treatment correlated with a shift in cell cycle phases and analysis of these showed that the presence of SDA-Col I markedly changed their distribution compared with control cells and upregulated the S phase ~1.8-fold at 5.0 *μ*g/*μ*L SDA-Col I (versus ~1.3-fold for 10 nM Dex).

Assessment of early osteogenic differentiation markers, ALP and OC, showed that these were increased nearly 1.4-fold compared with control cells. Increased expression of cellular ALP in distinct granular surface and perinuclear structures was observed to occur in concert with distinct morphological changes in BMSCs not observed under mock treatment, which suggests not only that these effects are linked, but also that SDA Col I is a causative factor. With increasing time, both proliferation and expression of ALP were reduced, whereas OC remained elevated, which indicates commitment to differentiation. However, high concentrations of SDA-Col I seemed to promote adipogenic differentiation, a phenomenon that is known to occur in the presence of Col I and resembles the action of Dex at particular concentrations [32, 40, 41] and is currently under further investigation. Since adipogenic differentiation of mesenchymal stem cells involves peroxisome proliferators activated receptor-*γ* (PPAR*γ*) and CCAAT/enhancer binding proteins (C/EBPs) [42], it would be worthwhile to investigate if these proteins are involved in SDA-Col I-induced adipogenesis. Furthermore, a major question that arises is how this switch from osteogenesis to adipogenesis occurs in the presence of SDA-Col I. One possibility might be that at higher concentrations, SDA-Col I is physically organized differently on the cell surface, thereby activating different receptors and signaling cascades [41, 43]. On the other hand, BMP-2 has been shown to be a key element in determining BMSC differentiation fate. One strategy to nullify SDA-Col I-induced adipogenesis and concomitantly promote (trans)differentiation of preadipocytes already present in bone, particular in osteoporosis treatment strategies, might be to combine SDA-Col I with retinoic acid (RA), since various forms of RA have been shown to repress adipogenic differentiation and cooperate with BMP2 and BMP9 in osteogenic (trans)differentiation in preadipocytes [44, 45]. Overall, our initial observations with SDA-Col I are in good agreement with various reports and even early* in vitro* work that demonstrated collagen type I-induced modulation of cell activities, including proliferation, differentiation, and mineralization [16, 46, 47].

Evaluation of key components of the BMP/Smad signaling pathway [45, 48, 49], the main bone morphogenetic cascade, showed that BMP2, Smad2, Smad3, and Smad4 were all increased by at least 30–45%. This shows the involvement of the Smad pathway during SDA-Col I-induced osteogenic differentiation of BMSCs, which was to be expected due to the prominent role that Smad signaling plays in both osteoblastic differentiation and chondrogenesis. Furthermore, ALP expression, which was significantly increased in response to SDA-Col I treatment, involves much of the same pathways, that is, the BMP/Runx2 (CBAf1, AML3)/Osterix system, and the Wnt signaling cascade [50, 51]. However, the current results do not provide sufficient evidence on the exact sequence of signaling events and pathways involved, which requires the scrutiny of key proteins in the BMP, TGF-*β*, and collagen receptor cascades. The involvement of integrins in collagen binding and signaling is well established, that is, the *α*_1_ *β*_1_, *α*_2_ *β*_1_, *α*_10_ *β*_1_, and *α*_11_ *β*_1_ integrins and downstream proteins [52]. Furthermore, Suzawa's group recently showed that collagen-induced FAK/ERK and BMP signaling congregate at the level of SMAD activation in the nucleus [18]. Because SDA-Col I in our formulation is present in a nonfibrillous form and consequently no long cellular projections were observed, we assume that *α*_1_ *β*_1_ integrin was predominantly involved. Indeed, only *α*_1_ *β*_1_ integrin has been shown to effectively bind type I collagen monomers [43] and at the same time stimulate proliferation [53, 54]. Furthermore, that the integrin-BMP/Smad signaling pathway in BMSCs is activated in response to both extracellular cues and the physical presence of collagen type I has been demonstrated when growing cells on nanofibrous hydroxyapatite/chitosan [55] or collagen I gel coated poly(lactic-co-glycolic)acid-*β*-tricalcium phosphate [56] scaffolds. Therefore, a further scrutiny of SDA-Col I binding to integrins, including evaluation of the differences between applications of SDA-Col I in solution compared with growing stem cells on SDA-Col I might not only provide more information on the osteogenic induction processes involved, but also allow optimization of potential treatment strategies. We are currently optimizing SDA-Col I-based liquid treatment modalities and investigating the effect of growing cells on SDA-Col I coated scaffolds.

In conclusion, our results generally demonstrate that SDA-Col I can induce BMSC proliferation and osteogenic differentiation, increase the expression of ALP and OC, and activate the BMP/Smad signaling pathways. The results from this study also underscore the importance of the physical presence of ECM proteins, well beyond their structural support role, but rather through ECM-mediated signalling in cells.

## Figures and Tables

**Table 1:** Overview of the PCR primers used.

Gene	Primer
Smad2 (380 bp)	5′-TTCCGCCTCTGGATGACTA-3′
	5′-TTTCTACCGTGGCATTTCG-3′

Smad3 (380 bp)	5′-GCACATAATAACTTGGACC-3′
	5′-CGTGTATTATTGAACCTGG-3′

Smad4 (380 bp)	5′-TTCTCCGAACGTGTCACGT-3′
	5′-AAGAGGCTTGCACAGTGCT-3′

OC (264 bp)	5′-GACACCATGAGGACCATCTT-3′
	5′-TTTTGGAGCTGCTGTGACAT-3′

ALP (298 bp)	5′-TTGGAAGAGCTTTAAACAG-3′
	5′-TGAAGGGCTTCTTGTCCGT-3′

*β*-actin (307 bp)	5′-TGAACGGGAAGCTCACTGG-3′
	5′-TCCACCACCCTGTTGCTGTA-3′

**Table 2:** Molecular size distributions with HPLC of collagen I.

Number	Retention time	Molecular weight	Peak area
	(min)	(kDa)	(Percentage %)
1	8.37	147.588	2.51
2	20.12	15.736	47.75
3	20.64	9.137	49.74

**Table 3:** Amino acid composition of collagen I.

	Retention time	Peak area	Peak area % (1)	Peak area % (2)	Content (%)
Asp	4.959	580.4	1.859	1.865	1.862
Glu	6.148	774.9	2.482	2.495	2.489
Hyp	8.766	2088.6	6.691	6.726	6.709
Ser	12.486	995.3	3.188	2.812	3.000
Gly	13.6	10020.9	32.197	32.006	32.102
His	17.519	74.3	0.238	0.234	0.236
Thr	21.431	601.9	1.928	1.9	1.914
Arg	22.418	2342.2	7.503	7.302	7.403
Ala	23.14	4284.1	13.724	14.005	13.865
Pro	24.457	4773.3	15.29	15.71	15.500
Tyr	29.67	120.4	0.386	0.381	0.384
Val	30.178	654.3	2.086	2.059	2.073
Met	31.389	85.8	0.275	0.269	0.272
Ile	33.953	248.2	0.795	0.795	0.795
Leu	34.149	934.4	2.993	2.950	2.972
Phe	35.085	613.7	1.966	1.926	1.946
Lys	35.744	1997.2	6.398	6.565	6.482
